# Rapid and Visual Detection of *Vibrio parahaemolyticus* in Aquatic Foods Using *bla*_CARB-17_ Gene-Based Loop-Mediated Isothermal Amplification with Lateral Flow Dipstick (LAMP-LFD)

**DOI:** 10.4014/jmb.2107.07022

**Published:** 2021-09-04

**Authors:** Yuan-qing Hu, Xian-hui Huang, Li-qing Guo, Zi-chen Shen, Lin-xue LV, Feng-xia Li, Zan-hu Zhou, Dan-feng Zhang

**Affiliations:** 1 School of Biological Science and Biotechnology, Minnan Normal University, Zhangzhou 363000, P.R. China; 2 Zhangzhou Center for Disease Control and Prevention, Zhangzhou 363000, P.R. China; 3 Comprehensive Technical Service Center, Zhangzhou Customs, Zhangzhou 363000, P.R. China

**Keywords:** *Vibrio parahaemolyticus*, loop-mediated isothermal amplification (LAMP), lateral flow dipstick (LFD), *bla*_CARB-17_ gene, rapid molecular detection, food safety

## Abstract

*Vibrio parahaemolyticus* is recognized as one of the most important foodborne pathogens responsible for gastroenteritis in humans. The *bla*_CARB-17_ gene is an intrinsic &beta;-lactamase gene and a novel species-specific genetic marker of *V. parahaemolyticus*. In this study, a loop-mediated isothermal amplification (LAMP) assay combined with a lateral flow dipstick (LFD) was developed targeting this *bla*_CARB-17_ gene. The specificity of LAMP-LFD was ascertained by detecting *V. parahaemolyticus* ATCC 17802 and seven other non-*V. parahaemolyticus* strains. Finally, the practicability of LAMP-LFD was confirmed by detection with *V. parahaemolyticus*-contaminated samples and natural food samples. The results showed that the optimized reaction parameters of LAMP are as follows: 2.4 mmol/l Mg^2+^, 0.96 mmol/l dNTPs, 4.8 U *Bst* DNA polymerase, and an 8:1 ratio of inner primer to outer primer, at 63°C for 40 min. The optimized reaction time of the LFD assay is 60 min. Cross-reactivity analysis with the seven non-*V. parahaemolyticus* strains showed that LAMP-LFD was exclusively specific for *V. parahaemolyticus*. The detection limit of LAMP-LFD for *V. parahaemolyticus* genomic DNA was 2.1 &times; 10^-4^ ng/μl, corresponding to 630 fg/reaction and displaying a sensitivity that is 100-fold higher than that of conventional PCR. LAMP-LFD in a spiking study revealed a detection limit of approximately 6 CFU/ml, which was similar with conventional PCR. The developed LAMP-LFD specifically identified the 10 *V. parahaemolyticus* isolates from 30 seafood samples, suggesting that this LAMP-LFD may be a suitable diagnostic method for detecting *V. parahaemolyticus* in aquatic foods.

## Introduction

*Vibrio parahaemolyticus* is a gram-negative, rod-shaped, motile, and non-spore-forming bacterium that is naturally present in estuarine and marine environments [1]. It has also evolved as the leading cause of seafood-associated gastroenteritis in humans [1, 2]. Abundance can be especially high in bivalve mollusks, which are filter-feeding animals that may accumulate this pathogen in their soft tissues, thus increasing the risk of transmission and infection of *V. parahaemolyticus* [3]. Due to the consumption of raw or undercooked aquatic products, humans can be infected by this pathogen during the lengthy farm-to-table process [4]. To ensure the supply of safe food and prevent economic losses, early monitoring and surveillance of *V. parahaemolyticus* are of utmost importance. Therefore, it is necessary to develop an easy-to-perform detection method with higher specificity and sensitivity.

Traditional methods of detecting *V. parahaemolyticus* in foods require labor-intensive and time-consuming cultures, and they usually include enrichment, separation and purification, microscopic examination, and biochemical testing [5, 6]. Moreover, not only do conventional culture methods take 72 h or longer, they also involve complicated steps that are strongly affected by this environmental microorganism as well as human error [6]. Recently, various PCR-based approaches, including conventional PCR, multiplex PCR, and real-time PCR, have been developed and used widely to detect *V. parahaemolyticus* from clinical, environmental, and seafood samples [7, 8]. Most of these methods usually require 2-3 days to complete the whole identification process [9]. Moreover, these techniques depend on sophisticated infrastructure and expensive reagents, which limits the implementation for onsite detection [10]. Recently, loop-mediated isothermal amplification (LAMP) has emerged as an alternative to PCR assays for the detection of *V. parahaemolyticus*. LAMP amplification occurs at a uniform temperature, so it requires only a small water bath or a simple heating block instead of thermocyclers [11, 12]. Significantly, the LAMP protocol can be completed within 1-2 h, which saves a lot of time [13].

LAMP is one of the most promising methods among the various new isothermal nucleic acid amplification techniques due to its high specificity, sensitivity, and convenience [14]. To detect LAMP products, several methods can be used, such as agarose gel electrophoresis (AGE), dsDNA-specific fluorescent dye, and turbidity of magnesium pyrophosphate [15]. Nevertheless, AGE experiment requires various instruments and materials and may be polluted by aerosols, which causes the false-positive determination in later assays [9, 16, 17]. To avoid the above disadvantages of monitoring LAMP amplicons, the lateral flow dipstick (LFD) was used as a tool to improve accuracy and reduce detection time. The LFD approach can correctly detect biotin-labeled LAMP products, which specifically hybridize with DNA probes conjugated with fluorescein isothiocyanate (FITC) and are identified by anti-FITC antibodies conjugated with nanogold [18]. This hybridization product is trapped by a biotin ligand and bound to a lateral flow test strip in the case of biotin-labeled LAMP amplicons, whereas non-hybridized digoxin-labeled probes are identified at another test line with goat anti-mouse immunoglobulin G (IgG) antibody [18]. The use of digoxin-labeled probes improves the detection sensitivity and ensures accuracy [18]. In addition, the LFD assay only takes 10-15 min, compared to 45-50 min required for the AGE experiment [19].

LAMP assays have proved to be a prominent tool for detecting seafood-borne pathogens, including *V. parahaemolyticus* in various aquatic foods [1, 20]. In previous studies, some key biomarkers, such as *tlh*, *tdh*, *trh*, *ompA*, and *toxR*, were selected as targets for *V. parahaemolyticus* detection [5, 20]. Although these targeting genes are species-specific markers, some genes may have analogous sequences within the *Vibrio* species [21], lowering the specificity of molecular identification methods. In 2016, a novel *bla*_CARB-17_-like gene was found closely associated with intrinsic penicillin resistance in *V. parahaemolyticus* [22, 23]. Li *et al*. studied the homologies of the *tlh*, *atpA*, and *bla*_CARB-17_ genes, and the latter was more highly conserved than the other two genes [22]. The PCR specificity in targeting the *bla*_CARB-17_ gene was 100%, whereas the PCR based on the *tlh* or *atpA* gene occasionally yielded false positives [22, 24, 25].

**Fig. 1:**
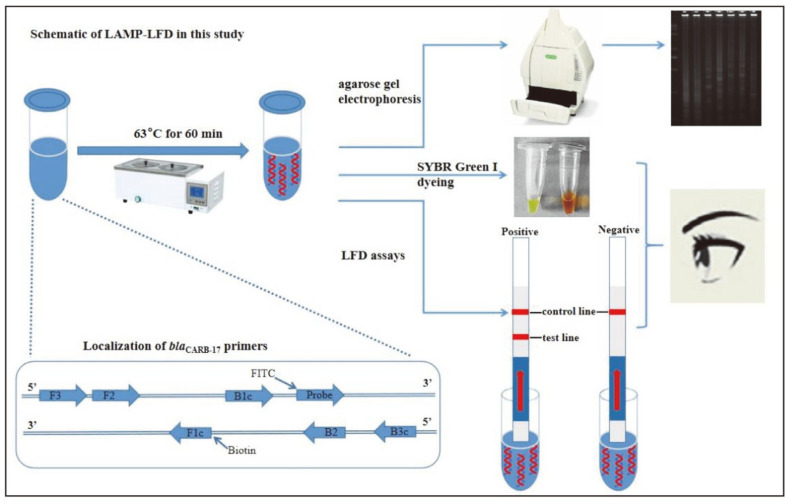
Schematic of LAMP-LFD based on *bla*_CARB-17_ gene in this study.

The present study aimed to develop a simple, efficient, and sensitive LAMP-LFD assay targeting the *bla*_CARB-17_ gene, a novel species-specific marker of *V. parahaemolyticus*. For this goal, three sets of LAMP primers and a FITC-labeled DNA probe were designed based on the *bla*_CARB-17_ sequence of *V. parahaemolyticus* ATCC 17802. Six significant parameters of LAMP reaction were optimized, LFD detection was combined with LAMP, and the sensitivity and specificity were assessed for the developed LAMP-LFD. Lastly, the practicability of the novel LAMP-LFD assay was evaluated utilizing *V. parahaemolyticus*-contaminated samples and natural food samples. A schematic of the LAMP-LFD based on *bla*_CARB-17_ gene is shown in Fig. 1.

## Materials and Methods

### Chemicals, Kit, and Bacterial Strains

Bst DNA polymerase (large fragment) and dNTPs were obtained from Tiangen Biotech (China). SYBR Green I and the EasyPure Genomic DNA Kit (GK1071) were procured from Generay Biotech Co., Ltd. (China). Primers used in the study were custom synthesized from Generay Biotech. Biowest agarose was obtained from Thermo Fisher Scientific (USA). A universal rapid dipstick kit made by Milenia Biotec GmbH (Milenia GenLine HybriDetect, Germany) was used to test the LAMP amplicons by LFD.

*V. parahaemolyticus* ATCC 17802 was employed to develop the LAMP-LFD assay. Pure culture stocks of *V. parahaemolyticus* were retrieved from the glycerol stock (-80°C) and cultured in thiosulfate-citrate-bile salts-sucrose (TCBS) agar plates at 37°C for 18-24 h. It was enriched by inoculation into 5 ml of alkaline peptone water (APW, Land Bridge Technology, China) containing 3% (w/v) NaCl. Seven non-*Vibrio parahaemolyticus* reference strains (Table 1) were used as control in this study. The strains of *Vibrio vulnificus* and *Staphylococcus aureus* were enriched in APW medium containing 3% (w/v) NaCl at 37°C for 18-24 h. The strains of *Pseudomonas aeruginosa*, *Proteus mirabilis*, *Candida albicans*, *Streptococcus pyogenes* and *Salmonella enteritidis* were enriched in APW medium at 37°C for 18-24 h.

### Genomic DNA Extraction

The genomic DNA of both *V. parahaemolyticus* and non-*Vibrio parahaemolyticus* strains was extracted using a TIANamp Bacteria DNA Kit (Tiangen Biotech Co., Ltd., China) according to the manufacturer’s instructions. In brief, 2 ml of bacterial cells was pelleted by centrifugation at 10,000 ×*g* for 2 min. For gram-positive bacteria, the pellet was re-suspended in 200 μl of buffer solution (GA) and incubated at 37°C for 40 min followed by the addition of 20 μl of proteinase K solution (20 mg/ml). For gram-negative bacteria, the pellet was re-suspended in 200 μl of buffer solution (GA). Then, the cells were lysed by the addition of 220 μl of lysis solution (GB) and incubation at 70°C for 10 min. The lysates were prepared by the addition of 220 μl of absolute ethanol. The cell lysates were transferred onto a Miniprep Spin column and centrifuged at 10,000 ×*g* for 30 s. The filtrate was discarded after washing with 500 μl of buffer solution (GD) and washing solution (PW) at 10,000 ×*g* for 1 min. Finally, the Miniprep Spin column was moved to a new collection tube, and 100 μl of elution buffer was added and incubated at room temperature for 2 min. The DNA solution was obtained after centrifuging at 10,000 ×*g* for 2 min and was checked for its concentration and purity using a NanoDrop One spectrophotometer (Thermo Fisher Scientific). The extracted DNA was stored at -80°C for further use.

### Design of LAMP and PCR Primers

**Fig. 2:**
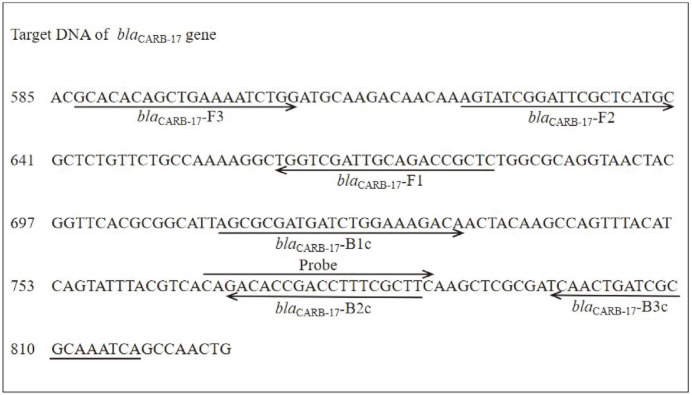
Schematic diagram of LAMP/LAMP-LFD primer and probe designed for the detection of *V. parahaemolyticus* ATCC17802. The arrows show the direction of primer extension and the binding sites of the primer sequences.

The primers were designed using Primer Explorer V4 software (http://primerexplore-r.jp/e/) based on the conserved sequence of *bla*_CARB-17_ gene (KJ934265) from *V. parahaemolyticus* ATCC 17802. In this study, the four LAMP primers consisted of a forward outer primer (F3), a backward outer primer (B3), a forward inner primer (FIP), and a backward inner primer (BIP), specifically designed to recognize the six distinct regions of the *bla*_CARB-17_ sequence. For LFD assays, one probe was designed based on the nucleotide sequence of the LAMP product and labeled at the 5'-end using fluorescein isothiocyanate (FITC). The 5'-end of the FIP primer was labeled using biotin (BIO). The schematic diagram of the LAMP-LFD primer and probe is shown in Fig. 2. All these primers were confirmed for their specificity with the BLASTn program, and oligonucleotides were synthesized by Sangon Biotech Co. (China).

### LAMP-LFD Assay and Reaction Condition Optimization

The preliminary LAMP assay was performed following the previous studies with some modifications [26]. In brief, the LAMP reaction was carried out in a volume of 25 μl, containing 2.4 μl MgSO_4_ (50 mmol/l), 3.6 μl dNTPs (10 mmol/l), 1 μl *Bst* DNA polymerase (8 U/μl), 1 μl of DNA template, 0.5 μl of each F3 (10 μmol/l) and B3 (10 μmol/l), 4 μl of each FIP (10 μmol/l) and BIP (10 μmol/l), and sterile double distilled water (ddH_2_O) to make up the volume. Reaction was incubated at 63°C for 60 min, and then the *Bst* DNA polymerase was inactivated at 80°C for 20 min. The LAMP products were analyzed using 2% agarose gel electrophoresis, and detected using 1.0 μl of 1/10-diluted original SYBR Green I dye, which produced a fluorescent green color in positive samples. On the basis of the above reaction conditions, optimization of the six parameters was carried out to determine the optimal LAMP conditions, including the concentration of magnesium ion (Mg^2+^) (1.2-7.2 mmol/l) and dNTP (0.64-1.76 mmol/l), the concentration ratio of the inner primer to the outer primer (4:1-12:1), the dosage of *Bst* DNA polymerase (large fragment) (0-8 U), reaction temperature (57-67°C), and time (20-70 min) (Table 2).

For the LFD assays, the reactions were performed by using 20 pmol/l of the FIP primer labeled with FITC, and the same concentration of unlabeled F3, B3, and BIP primers as the LAMP. After the LAMP step, the mixture was added using 20 pmol/l of the probe labeled with BIO for further hybridization reaction at 63°C for 5 min. Then, the dipstick was placed into a mixture of 80 μl of HybriDetect buffer and 5 μl of hybridizing solution for 3 min. The results were scored as positive when both the test line and the control line were displayed and as negative when only the control line was visible. On the basis of the upper conditions of LFD, optimization of the shortest amplification time was conducted at 63°C for 20, 40, and 60 min, respectively.

### Determination of Specificity and Sensitivity of LAMP-LFD

To investigate the specificity of the developed LAMP-LFD, the genomic DNA was prepared from strains of *V. parahaemolyticus* and non-*V. parahaemolyticus* as mentioned above. Using these DNA solutions as templates, the cross-reactivity was checked using conventional PCR as controls. The PCR was carried out using F3 and B3 as primers to amplify a 220 bp fragment. The 25 μl of the reaction mixture for the PCR assay was composed of 12.5 μl of 2×Taq Master Mix (Shanghai Generay Biotech Co., Ltd., China), 1 μM of each primer (F3 and B3), 3 μl of the DNA template, and the remaining volume of ddH_2_O. The thermal-cycling program is as follows: initial denaturation at 95°C for 5 min, followed by 30 cycles of 95°C for 30 s, 58°C for 30 s and 72°C for 30 s, and a final extension of 72°C for 5 min. PCR amplification was run on a Bio-Rad PTC-200 Thermal Cycler (Bio-Rad, USA). The amplified products were analyzed electrophoretically on a 2% agarose gel containing Gel Stain (Sangon Biotech, China) and photographed using an Amersham Imager 600 UV (GE Healthcare, USA). The LAMP-LFD reaction was performed under optimal conditions, and the DNA solution was replaced with ddH_2_O in the negative control.

To determine the sensitivity, serial dilutions (1:100) of the genomic DNA of *V. parahaemolyticus* were used for the evaluation of the LAMP-LFD assay in comparison with conventional PCR. Briefly, *V. parahaemolyticus* genomic DNA was diluted as a serial concentration (2.1 × 10^2^-2.1×10^-4^ ng/μl) using nuclease-free ddH_2_O. Both LAMP-LFD and PCR were performed using all these dilutions as templates, and ddH_2_O was used to replace DNA as template for the negative control. Products of LAMP were analyzed using 2% agarose gel electrophoresis, SYBR Green I, and dipstick. The PCR products were analyzed with 2% agarose gel electrophoresis.

### Artificial Spiking Study and Limit of Detection

To further evaluate the practicability of the developed LAMP-LFD method, the limit of detection (LOD) was determined by using *V. parahaemolyticus*-contaminated samples as an aquatic food model [10, 27], with PCR and SYBR Green I methods as controls. In brief, fresh shrimp were procured from the local market of Zhangzhou, Fujian Province, China, and confirmed to be free from *V. parahaemolyticus* through conventional culture and PCR assays. Shrimp meat was homogenized in APW with 3% NaCl. *V. parahaemolyticus* (ATCC 17802) was cultured in APW with 3% NaCl at 37°C overnight and centrifuged at 5,000 ×*g* for 5 min. The bacterial cells were washed using sterile 1×phosphate-buffered saline (PBS, 137 mM NaCl, 10 mM Na_2_HPO_4_, 1.8 mM KH_2_PO_4_, and 2.7 mM KCl; pH7.4) and re-suspended in 10 ml of sterile 1×PBS. Serial dilutions (6 × 10^7^ CFU/ml to 6×10^-3^ CFU/ml) of bacterial cells were prepared with sterile 1×PBS. The bacterial concentration in the stock culture was estimated by spreading 100 μl of serial dilutions onto the TCBS agar plate in duplicate followed by incubation at 37°C overnight in an incubator. One milliliter of each serial dilution was mixed with 9 ml of the meat homogenate dispensed in 15 ml culture tubes. The negative control was maintained by inoculating the shrimp homogenate with 1 ml sterile 1×PBS. Genomic DNA was prepared from each dilution of inoculated meat using the TIANamp Bacteria DNA Kit (Tiangen Biotech Co., Ltd.), and both LAMP and PCR were performed with the extracted genomic DNA.

### Detection of *V. parahaemolyticus* in Natural Food Samples Using LAMP-LFD

Shellfish products were utilized to evaluate the reliability of the LAMP-LFD assays in natural food samples: 30 aquatic products (short-necked clam, *n* = 10; Asiatic hard clam, *n* = 10; razor clam, *n* = 10) were collected from 3 local retail markets in Zhangzhou, China. *V. parahaemolyticus* isolates were detected from natural food samples following the method described by Yuanqing [28]. Briefly, 25 g of each sample was homogenized with 225 ml alkaline peptone water (APW, Land Bridge Technology, China) containing 3% (w/v) NaCl, and homogenates were streaked onto TCBS agar plates and incubated at 37°C for 18-24 h. Green or blue green colonies were spread onto new TCBS plates for purification at 37°C for 18 h, and presumptive colonies were chosen for identification by Gram staining and biochemical tests (oxidase activity; halophilic test; urease test; citrate utilization test; decomposition test of sucrose, glucose and mannitol; 3% (w/v) NaCl triple sugar iron tests). Presumptive *V. parahaemolyticus* isolates were confirmed using PCR for detecting the *bla*_CARB-17_ gene [22]. The primers were *bla*_CARB-17_-F (5'-ACYTTGATGGAAGATA-3') and *bla*_CARB-17_-R (5'-YTAACTTTCTTTGTAGTGM-3') [22]. For LFD assays, the food samples were pre-enriched and DNA was extracted using the TIANamp Bacteria DNA Kit (Tiangen Biotech Co., Ltd., China) and following the manufacturer’s instructions for the detection of *V. parahaemolyticus*.

## Results

### Optimization of LAMP Assay Conditions

**Fig. 3:**
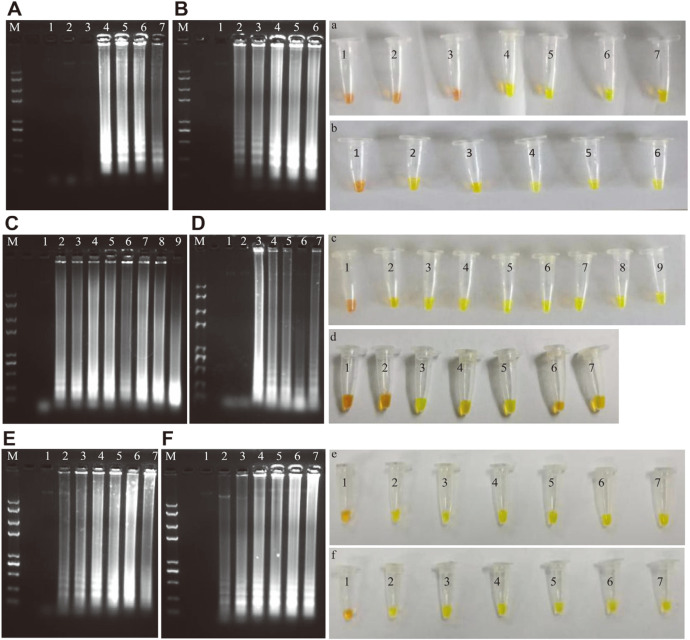
Optimization of LAMP conditions, including the concentrations of dosage of *Bst* DNA polymerase (A, a), the concentration ratio of inner primer to outer primer (B, b), the concentration of dNTP (C, c), the concentration of Mg^2+^ (D, d), reaction temperature (E, e), reaction time (F, f). M, *Trans* 2K Plus II DNA Marker. A and a Lanes/Tubes 2-7: the dosage of *Bst* DNA polymerase is 0, 1.6, 3.2, 4.8, 6.4, 8 U, respectively, Lane/Tube 1 is negative control; B and b Lanes/Tubes 2-6: the ratio of inner primer to outer primer is 4:1, 6:1, 8:1, 10:1, 12:1, respectively, Lane/Tube 1 is negative control; C and c Lanes/Tubes 2-9: the concentration of dNTP is 0.64, 0.8, 0.96, 1.12, 1.28, 1.44, 1.6, 1.76 mmol/l, respectively, Lane/Tube 1 is negative control; D and d Lanes/Tubes 2-7: the concentration of Mg^2+^ is 1.2, 2.4, 3.6, 4.8, 6.0, 7.2 mmol/l, respectively, Lane/Tube 1 is negative control; E and e Lanes/Tubes 2-7: the reaction temperature is 57, 59, 61, 63, 65, 67°C, respectively, Lane/Tube 1 is negative control; F and f Lanes/Tubes 2-7: the reaction time is 20, 30, 40, 50, 60, 70 min, respectively, Lane/Tube 1 is negative control.

The LAMP reaction was performed in a 25 μl mixture, and the optimized reactive system included 1×*Bst* buffer, 2.4 mmol/l MgSO_4_, 1.6 mmol/l dNTP, 4.8 U *Bst* DNA polymerase, a concentration ratio of inner primer-outer primer (8:1), 1 μl of template genomic DNA, and nuclease-free ddH_2_O to make up the volume (Fig. 3). The reaction was incubated at 63°C for 60 min for amplification, and then the enzymatic activity was inactivated at 80°C for 5 min. LAMP products were analyzed by the addition of SYBR Green I dye where positive results were indicated by a change from orange to fluorescent green, whereas in negative reactions, the orange color was mostly conserved (Figs. 3A-3F). When the LAMP products were subjected to agarose gel electrophoresis, the results showed a ladder pattern due to formation of stem-loop structures of different lengths (Figs. 3A-3F).

### Development of LAMP-LFD and Optimization of Reaction Time

**Fig. 4:**
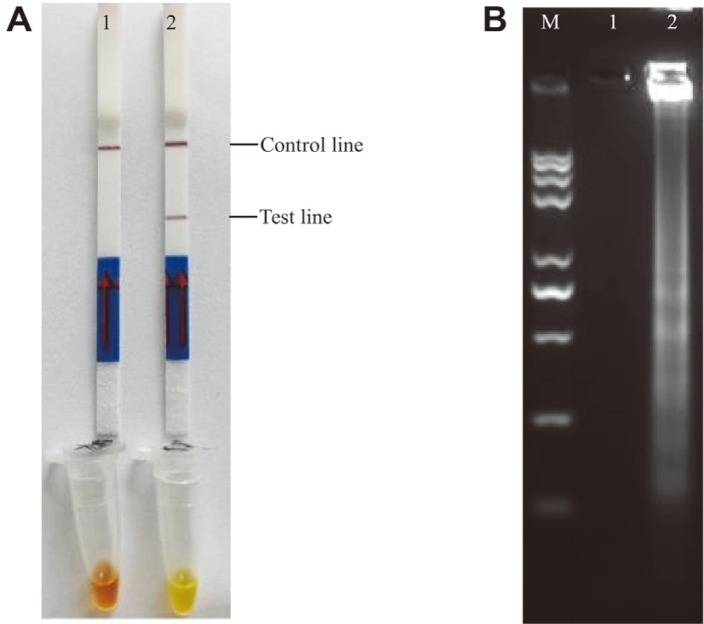
Development of LAMP-LFD reaction. A and B Lanes/Tubes 1: the negative control (ddH_2_O); Lanes/Tubes 2: the DNA is from *Vibrio parahaemolyticus* ATCC17802.

Under the optimized reaction conditions of LAMP, the successful LFD assay was developed with the probe oligonucleotide and FIP primer labeled at the 5'-end with FITC and BIO, respectively. LAMP-LFD and SYBR Green I colorimetric detection simultaneously produce dual lines (control line and test line) and a visible green reaction (Fig. 4A). Moreover, LAMP-AGE could yield bright electrophoresis bands (Fig. 4B). The results were clearly negative when ddH_2_O was used as a blank control (Fig. 4B).

**Fig. 5:**
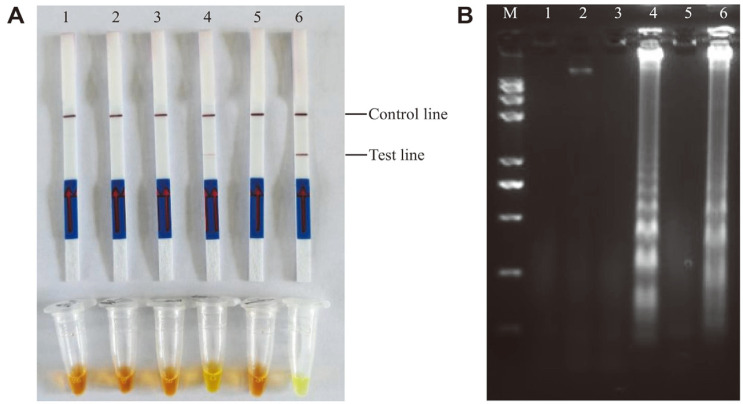
Optimization of LAMP-LFD reaction time. **A** and **B** Lanes/Tubes 1-2: the reaction time is 20 min, Lanes/Tubes 3-4: the reaction time is 40 min, Lanes/Tubes 5-6: the reaction time is 60 min. **A** and **B** Lanes/Tubes 1, 3, 5: the negative control (ddH_2_O); Lanes/Tubes 2, 4, 6: the DNA from *Vibrio parahaemolyticus* ATCC17802.

To determine the minimal LFD assay time, three different points of 20, 40, and 60 min for incubation were examined in a water bath (63°C). When the reaction was 40 min, the results of SYBR Green I dye and LAMP-AGE could be detected narrowly, but dual lines were difficult to distinguish (Figs. 5A and 5B). When the reaction time was 60 min, three positive results could be observed at the same time (Figs. 5A and 5B). Therefore, the best reaction time was 60 min, and valuable results from the LFD assay could be obtained.

### Specificity Evaluation of LAMP-LFD

**Fig. 6:**
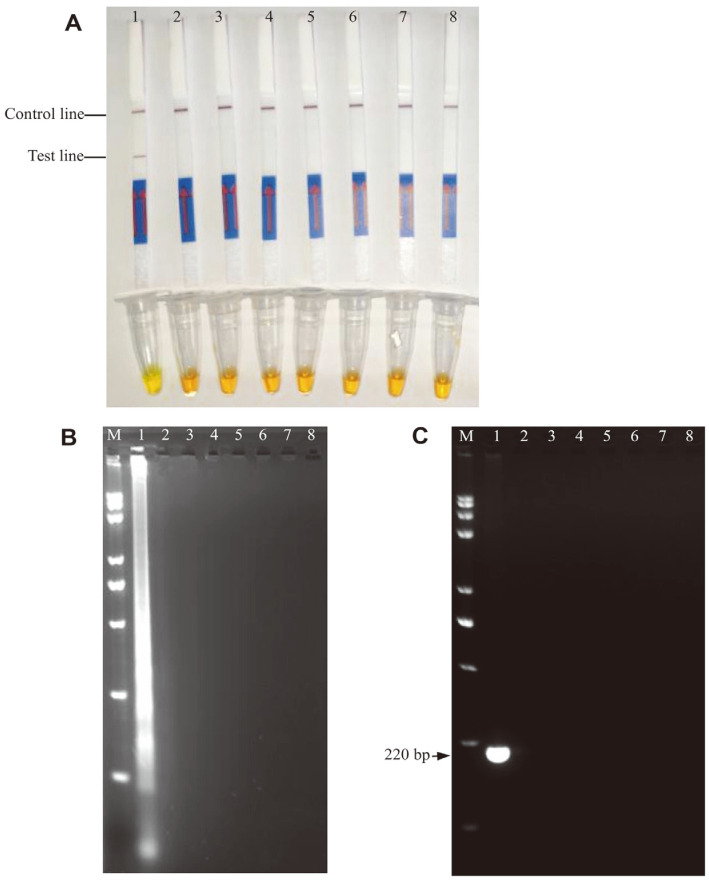
Specificity test of LAMP-LFD. A and B Lanes/Tubes 1-8: the DNA is from *Vibrio parahaemolyticus* ATCC17802, *Pseudomonas aeruginosa* ATCC27853, *Proteus mirabilis* ATCC33583, *Vibrio vulnificus* ATCC27562, *Salmonella enterica* ser. Enteritidis 50041, *Candida albicans* ATCC10231, *Streptococcus pyogenes* ATCC19615, *Staphylococcus aureus* CMCC26003, respectively. C is the results of PCR for 8 foodborne pathogens, 1-8: the DNA is from 8 pathogens in the same order as above.

The developed LAMP-LFD assay for *V. parahaemolyticus* targeting the *bla*_CARB-17_ gene confirmed a high degree of specificity due to the amplified products detected with genomic DNA of only *V. parahaemolyticus* ATCC 17802 as indicated by LAMP-LFD (Fig. 6A). In contrast, the other seven genomic DNA samples from foodborne pathogens were not detected by the dual-labeled amplicons with the LFD dipsticks. The same results could be obtained from 2% agarose gel electrophoresis (Figs. 6B and 6C) and SYBR Green I dye detection (Fig. 6A). In detail, bright green fluorescence was seen due to the binding of SYBR Green I dye to the stacked base of the amplified DNA fragments, while the orange color was maintained by the SYBR Green I dye if there was no amplification generated in the case of non-*V. parahaemolyticus* bacterial strains. Agarose gel electrophoresis of the LAMP reaction displayed a ladder pattern only with *V. parahaemolyticus* strains, while no amplified bands were observed in non-*V. parahaemolyticus* strains.

### Sensitivity Determination of LAMP-LFD

**Fig. 7:**
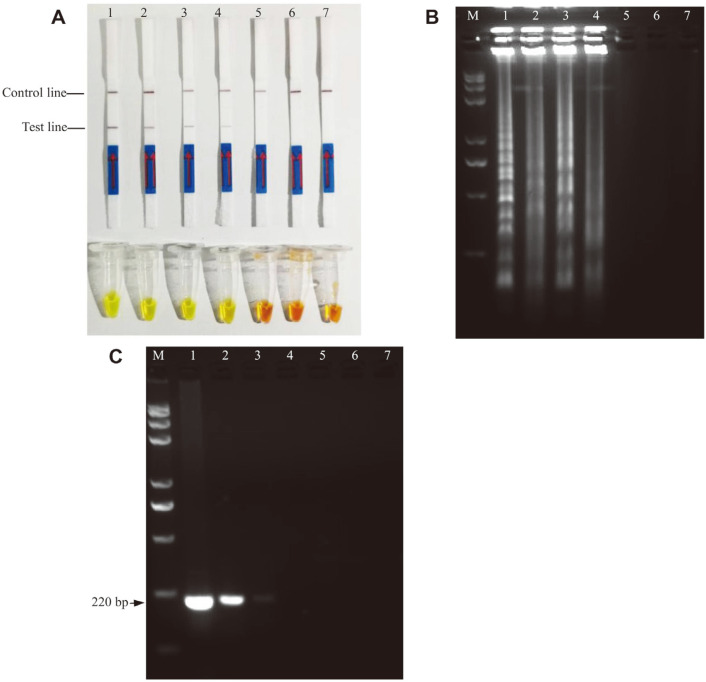
Sensitivity test of LAMP-LFD. A is the sensitivity of *Vibrio parahaemolyticus* by LFD and SYBR Green I; B is the result of sensitivity using electrophoresis; C is the result of conventional PCR. A, B, and C Lanes/Tubes 1-6: The DNA concentrations of *Vibrio parahaemolyticus* is 211.1 ng/μl, 2.1 ng/μl, 2.1 × 10^-2^ ng/μl, 2.1 × 10^-4^ ng/μl, 2.1 × 10^-6^ ng/μl, 2.1 × 10^-8^ ng/μl, respectively. Lanes/Tubes 7 is negative control (ddH_2_O).

The genomic DNA of *V. parahaemolyticus* ATCC17802 was used as a template to perform LAMP-LFD in comparison with SYBR Green I dye, AGE of LAMP products, and the conventional PCR assay (Fig. 7). A concentration of 211.1 ng/μl was extracted. Using 100-fold serial dilutions of genomic DNA, the concentrations ranged from 211.1 to 2.1 × 10^-8^ ng/μl. LAMP-LFD, SYBR Green I, and the LAMP-AGE assay displayed equal sensitivity of 2.1×10^-4^ ng/μl (Figs. 7A and 7B). In contrast, the PCR assay could only produce detectable electrophoresis bands with a concentration of 2.1 ng/μl, indicating LAMP-LFD to be 100-fold more sensitive than the PCR assay (Fig. 7C). After conversion, the detection limit of LAMP-LFD was 630 fg/reaction (3 μl of DNA/reaction).

### Detection of *V. parahaemolyticus*-Contaminated Samples Using LAMP-LFD

**Fig. 8:**
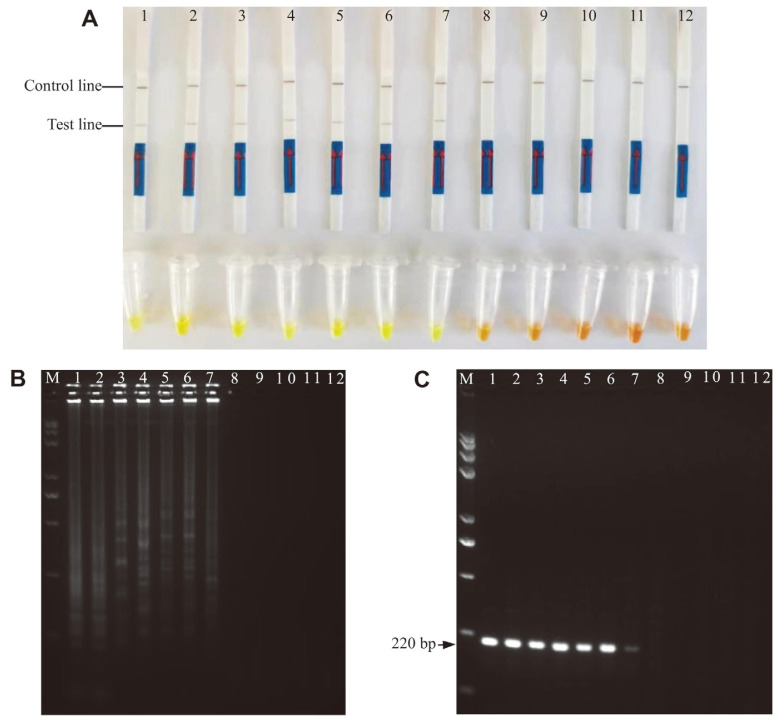
Analysis of *V. parahaemolyticus*-contaminated samples using developed LAMP-LFD. A is the result of *V. parahaemolyticus*-contaminated samples by LFD and SYBR Green I; B is the result of *V. parahaemolyticus*-contaminated samples using electrophoresis; C is the result of *V. parahaemolyticus*-contaminated samples using conventional PCR. A, B and C Lanes/Tubes 1-11: The cell concentrations of *Vibrio parahaemolyticus* are 6 × 10^6^ CFU/ml, 6 × 10^5^ CFU/ml, 6 × 10^4^ CFU/ml, 6 × 10^3^ CFU/ml, 6 × 10^2^ CFU/ml, 6 × 10^1^ CFU/ml, 6 × 10^0^ CFU/ml, 6 × 10^-1^ CFU/ml, 6 × 10^-2^ CFU/ml, 6 × 10^-3^ CFU/ml, 6 × 10^-4^ CFU/ml, respectively. Lanes/Tubes 12 is negative control (ddH_2_O).

The comparative results of LAMP-LFD, SYBR Green I, LAMP-AGE, and the conventional PCR assay for the *V. parahaemolyticus*-contaminated samples with 6 × 10^6^ CFU/ml to 6 × 10^-4^ CFU/ml of *V. parahaemolyticus* ATCC17802 are shown in Figs. 8A, 8B, and 8C. Positive LAMP-LFD could occur with the cell density ranging from 6×10^6^ CFU/ml to 6 CFU/ml, and SYBR Green I results producing a visible green reaction were observed in the same concentration range (Fig. 8A). Similarly, at this concentration, LAMP-AGE (Fig. 8B) and PCR-AGE (Fig. 8C) could produce detectable electrophoresis bands. In brief, no difference in the sensitivity of LAMP-LFD and the other assays was found in the spiking experiment, and the LOD was 6 CFU/ml for the established LAMP-LFD.

### LAMP-LFD Assays in Food Samples

**Fig. 9:**
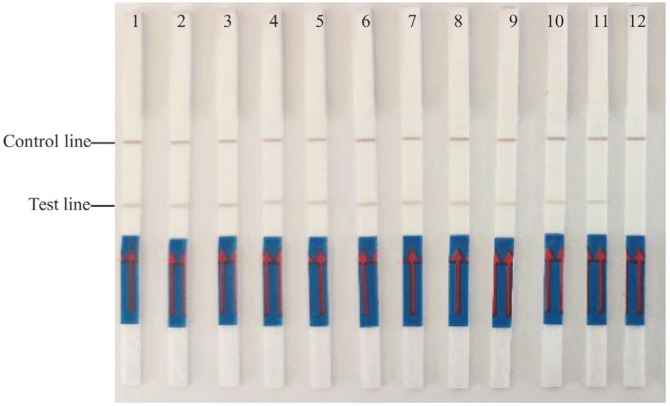
Analysis of aquatic food samples using developed LAMP-LFD. Lane 1 and Lane 12 are positive control (*Vibrio parahaemolyticus* ATCC17802) and negative control (ddH_2_O), respectively. Lanes 2-11: Ten natural samples were detected as positive using PCR.

A total of 30 aquatic products were collected as natural food samples, of which 10 isolates were detected to be *bla*_CARB-17_-positive using PCR as identified using the optimized LAMP-LFD method (Fig. 9). The accuracy was 100% between the newly developed LAMP-LFD method and the conventional PCR assay (results not shown).

## Discussion

*V. parahaemolyticus* is the most frequent cause of gastroenteritis among the 12 *Vibrio* species known to be pathogenic to humans [29]. The worldwide presence of *V. parahaemolyticus* in the marine environment raises constant concern regarding food safety in view of it likely causing disease outbreaks according to environmental conditions [7]. Owing to the growth of seafood consumption around the world, the output of aquatic products has dramatically increased in recent years. Hygiene problems with aquatic foods must be particularly highlighted during the seafood harvesting period from farm to fork, and there is an indispensable need to supervise the pathogen by means of simple, accurate, sensitive, high-throughput, and easy-to-use molecular biological techniques [1]. LAMP can achieve DNA synthesis of 10^6^-10^9^ copies within 30-60 min under isothermal conditions of 60 to 65°C [30], which can be provided using a disposable pocket warmer placed in a box [31]. LFD assays allow direct monitoring of LAMP amplicons by the naked eye [18]. The design and label of primers play an essential role in LAMP-LFD reaction [10]. In our previous study, two sets of primers were designed and synthesized for LAMP, and false-positive results were observed. A new set of primers was used for correct LAMP amplification in the present study. Referring to other studies with LAMP [17, 32], one probe was designed and labeled using fluorescein isothiocyanate (FITC), and the FIP primer was labeled using biotin (BIO) at the 5'-end.

The proper selection of a target gene for microorganism detection is significant for the specificity and accuracy of analytical techniques based on nucleic acid sequences [21]. Several genes, such as *tlh* [17, 32, 33], *toxR* [17], and 16-23S rRNA [17], have been developed to detect *V. parahaemolyticus* by the LAMP-LFD assay. Although *tlh* and *toxR* are present in *V. parahaemolyticus* strains at the 100% level, other *Vibrio* species also contain these two species-specific markers [17]. The *bla*_CARB-17_ gene is responsible for the intrinsic resistance to penicillins in *V. parahaemolyticus* [22, 23]. A comparative study was carried out to compare the specificity of PCR methods targeting *tlh*, *atpA*, *toxR*, and *bla*_CARB-17_ genes [22]. The assays detecting *toxR* and *bla*_CARB-17_ genes showed similar specificity, while the assays targeting *atpA* and *tlh* showed less specificity due to accidental false-positive results [22]. Phylogenetic analysis of these genetic markers showed that the *bla*_CARB-17_ gene for *V. parahaemolyticus* has the lowest degree of homology (78%) with V. alginolyticus in comparison with the *atpA* (97%), *toxR* (86%), and *tlh* (85%) genes [22, 34]. Thus, the *bla*_CARB-17_ gene is considered as a novel species-specific genetic marker that can be used as a reliable detection target in the future [22, 34]. Based on the above considerations, the *bla*_CARB-17_ gene was selected as a novel target to establish a more specific LAMP-LFD method in this study. The results suggested that the *bla*_CARB-17_ gene can be applied as a reliable molecular marker in LFD assays.

To our knowledge, the present study is the first report of LAMP and LAMP-LFD assays specifically based on the *bla*_CARB-17_ gene for detecting *V. parahaemolyticus* in aquatic foods. The reaction conditions and systems of LAMP-LFD, such as Mg^2+^ concentration, primer concentration, and enzyme concentration, can directly affect the sensitivity and specificity of the detection [35]. In our study, the optimization of LAMP-LFD conditions was first comparatively achieved under different concentrations of Mg^2+^ and dNTP, concentration ratios of the inner primer to the outer primer, *Bst* DNA polymerase quantity, and reaction temperature and time, since these parameters may influence the amplification efficiency of the LAMP-LFD assay [10]. The amount of *Bst* DNA polymerase, concentration of Mg^2+^, and reaction temperature are the key conditions of LAMP; the latter two can also act on the catalytic reaction by affecting the activity of *Bst* DNA polymerase [26, 36]. An appropriate amount of *Bst* DNA polymerase can ensure a stable reaction, and a higher concentration of *Bst* DNA polymerase not only increases the cost of the reaction but also makes the primer form a polymer and reduce the reaction efficiency [37]. A high concentration of Mg^2+^ also leads to instability of the system [26]. Temperature also affects the LAMP reaction by controlling enzyme activity: too high temperature will reduce the binding efficiency between primers and target fragments, and too low temperature will cause primers to form polymers and false positives [37, 38]. The internal primer is the decisive factor in the LAMP reaction [26, 38]. It takes the lead in complementary pairing with the target sequence and starts a chain extension under the action of *Bst* DNA polymerase [38]. The outer primer pairs with the target sequences according to free collision theory [26]. A high concentration of the outer primer can improve the efficiency of anchoring the target genes and provide the skeleton for the extension of the inner primer [26, 38]. The appropriate concentration of dNTPs is the raw-material guarantee for amplification reaction, which should not be excessive in the experiment, because a low concentration of dNTPs can reduce the wrong incorporation of nucleotides during non-target initiation and extension [11, 12, 26].

The optimized LAMP-LFD assay for *V. parahaemolyticus* could be finished within about 70 min and requires only a simple water bath to provide an isothermal condition at 63°C. Most molecular detection methods including conventional PCR, real-time PCR, and LAMP-AGE for detecting *V. parahaemolyticus* require at least 90 min [30]. In the current study, the results of the LAMP-LFD assay can easily be recognized by the naked eye within only 70 min. The results of LAMP can be analyzed by testing the white deposit, AGE, or visualization using SYBR Green I [39]. However, the green coloration and white precipitate are frequently too weak to distinguish by the naked eye [16, 17]. Moreover, aerosols can sometimes cause cross-contamination during AGE operations or SYBR Green I dyeing, thus false-positive amplification can be observed [11, 30]. LAMP combined with LFD method can greatly increase detection specificity, since the LFD dipstick truly detects dual-labeled LAMP products [40]. In addition, the LFD assay was more convenient than PCR, as test signals were directly visible to the naked eye from the dipstick. In the present study, the results suggest that the optimized LAMP-LFD assay was more specific, sensitive, and time saving.

The variability in the reported sensitivity of LAMP may be on account of differences in the location of primers selected by different researchers. Here, the sensitivity of the developed *bla*_CARB-17_-based LAMP-LFD assay was 210 fg/μl for pure genomic DNA and 6 CFU/ml for the *V. parahaemolyticus*-contaminated samples, which is evidently higher than that reported in the published literature amounting to 41 CFU/reaction [32], 430 CFU/ml [15], 10 pg/μl [41], and 92 CFU/reaction [42]. The current LAMP-LFD assay showed better sensitivity than that of the *bla*_CARB-17_-PCR method and was up to 100-fold more sensitive. This suggests that this new LAMP-LFD assay is more sensitive than the conventional PCR method. Because of the different amplification efficiencies of the designed primer sets, LAMP assays may show various sensitivities by using diverse targets or even the same target gene [30]. In addition, different optimized amplification conditions and amounts of added DNA templates can also bring about dissimilar results using LAMP-LFD [10, 30].

In this study, the applicability of the developed LAMP-LFD method was evaluated by an artificial spiking study and a natural food test. When the optimized LAMP-LFD method was applied to *V. parahaemolyticus*-contaminated samples, the LOD was 6 CFU/ml without any enrichment, which was comparable with the results of other LAMP-associated methods [43, 44]. For screening practical foods, 30 samples were collected and detected using a culture-based method and PCR. This new LAMP-LFD method exhibited 100% accuracy in comparison with the results of PCR, which suggests that the established LAMP-LFD method has better specificity and practicability than that of PCR without false-positive or false-negative results for the detection of *V. parahaemolyticus* in natural food samples. In summary, the development of this LAMP-LFD assay to detect *V. parahaemolyticus* provides a functional tool and paves the way for the establishment of a clinical kit, which can be applied as a new *V. parahaemolyticus* monitoring method in basic-level aquatic farming units.

## Supplemental Materials

Supplementary data for this paper are available on-line only at http://jmb.or.kr.

## Figures and Tables

**Table 1:** List of bacterial strains used for LAMP/LAMP-LFD assay.

Species	Strains	LAMP/LAMP-LFD test
*Vibrio parahaemolyticus*	ATCC17802	+
*Pseudomonas aeruginosa*	ATCC27853	-
*Proteus mirabilis*	ATCC33583	-
*Vibrio vulnificus*	ATCC27562	-
*Candida albicans*	ATCC10231	-
*Streptococcus pyogenes*	ATCC19615	-
*Salmonella enteritidis*	50041	-
*Staphylococcus aureus*	CMCC26003	-

**Table 2:** Design of parameter optimization in LAMP system.

Reaction conditions/unit	Parameters （increasing order）
*Bst* DNA polymerase/U	0, 1.6, 3.2, 4.8, 6.4, 8
Concentration ratio of inner primer to outer primer	4:1, 6:1, 8:1, 10:1, 12:1
Concentration of dNTP/mmol/l	0.64, 0.8, 0.96, 1.12, 1.28, 1.44, 1.6, 1.76
Concentration of Mg^2+^/mmol/l	1.2, 2.4, 3.6, 4.8, 6.0, 7.2
Reaction temperature/°C	57, 59, 61, 63, 65, 67
Reaction time/min	20, 30, 40, 50, 60, 70
