# Supplementary material for: Rapid and Visual Detection of Vibrio parahaemolyticus in Aquatic Foods Using blaCARB-17 Gene-Based Loop-Mediated Isothermal Amplification with Lateral Flow Dipstick (LAMP-LFD)
Source: J Microbiol Biotechnol. 2021 Sep 4;31(12):1672–83. doi: 10.4014/jmb.2107.07022 (PMC9705909; doi:10.4014/jmb.2107.07022)
Supplement: Supplementary file 1 [file jmb-31-12-1672-supple.pdf]

J. Microbiol. Biotechnol.
https://doi.org/10.4014/jmb.2107.07022

**Supplementary table 1. The information of 10 positive samples detected by LAMP-LFD**

| No. of samples | Samples | Sampling location | Sampling time |
|---|---|---|---|
| 1 | short-necked clam | Longhai, Zhangzhou City | January 2019 |
| 2 | Asiatic hard clam | Yunxiao, Zhangzhou City | February 2019 |
| 3 | razor clam | Yunxiao, Zhangzhou City | January 2019 |
| 4 | short-necked clam | Longhai, Zhangzhou City | April 2019 |
| 5 | razor clam | Zhangpu, Zhangzhou City | April 2019 |
| 6 | Asiatic hard clam | Zhao'an, Zhangzhou City | May 2019 |
| 7 | razor clam | Zhao'an, Zhangzhou City | February 2019 |
| 8 | short-necked clam | Longhai, Zhangzhou City | January 2019 |
| 9 | razor clam | Zhangpu, Zhangzhou City | May 2019 |
| 10 | Asiatic hard clam | Zhangpu, Zhangzhou City | July 2019 |

The number of samples corresponds to Lane 2-11 in Figure 9.
